# Phenobarbital Induces Alterations in the Proteome of Hepatocytes and Mesenchymal Cells of Rat Livers

**DOI:** 10.1371/journal.pone.0076137

**Published:** 2013-10-24

**Authors:** Philip Klepeisz, Sandra Sagmeister, Verena Haudek-Prinz, Melanie Pichlbauer, Bettina Grasl-Kraupp, Christopher Gerner

**Affiliations:** 1 Department of Inner Medicine I, Comprehensive Cancer Center, Institute of Cancer Research, Medical University of Vienna, Vienna, Austria; 2 Institute of Analytical Chemistry, Faculty of Chemistry, University of Vienna, Vienna, Austria; university of birmingham, United Kingdom

## Abstract

Preceding studies on the mode of action of non-genotoxic hepatocarcinogens (NGCs) have concentrated on alterations induced in hepatocytes (HCs). A potential role of non-parenchymal liver cells (NPCs) in NGC-driven hepatocarcinogenesis has been largely neglected so far. The aim of this study is to characterize NGC-induced alterations in the proteome profiles of HCs as well as NPCs. We chose the prototypic NGC phenobarbital (PB) which was applied to male rats for a period of 14 days. The livers of PB-treated rats were perfused by collagenase and the cell suspensions obtained were subjected to density gradient centrifugation to separate HCs from NPCs. In addition, HCs and NPC isolated from untreated animals were treated with PB *in vitro*. Proteome profiling was done by CHIP-HPLC and ion trap mass spectrometry. Proteome analyses of the *in vivo* experiments showed many of the PB effects previously described in HCs by other methods, e.g. induction of phase I and phase II drug metabolising enzymes. In NPCs proteins related to inflammation and immune regulation such as PAI-1 and S100-A10, ADP-ribosyl cyclase 1 and to cell migration such as kinesin-1 heavy chain, myosin regulatory light chain RLC-A and dihydropyrimidinase-related protein 1 were found to be induced, indicating major PB effects on these cells. Remarkably, *in vitro* treatment of HCs and NPCs with PB hardly reproduced the proteome alterations observed *in vivo*, indicating differences of NGC induced responses of cells at culture conditions compared to the intact organism. To conclude, the present study clearly demonstrated that PB induces proteome alterations not only in HCs but also in NPCs. Thus, any profound molecular understanding on the mode of action of NGCs has to consider effects on cells of the hepatic mesenchyme.

## Introduction

Screening assays, which enable the early detection of potential carcinogenic activities, are of crucial importance for safe drug development strategies. Chemical compounds may cause cancer by directly affecting DNA and are thus called genotoxic carcinogens. This type of compounds is easily detectable as such by the application of well-established *in vitro* assays, such as Ames bacterial reverse mutation assay, mammalian forward mutation assays and detection of chromosomal aberrations. Furthermore, *in vivo* assays are routinely used which include rodent erythrocyte micronucleus assay, mammalian bone marrow chromosomal aberration assay and assays for somatic cell gene mutation in endogenous genes. Chemical carcinogens which do not affect DNA directly are called non-genotoxic carcinogens (NGCs) [1]. In contrast to genotoxic carcinogens, there are no sufficiently accurate and validated short-term assays that may allow detection of NGCs [2–6]. Currently employed assays necessitate long-term rodent carcinogenicity assays causing high efforts, costs and time requirement as major drawbacks. In order to overcome these problems, deeper insights into NGC-relevant mechanisms are urgently required, which may be obtained by the application of a screening technology such as proteome profiling. 

According to current knowledge, a characteristic effect of many NGCs is a deviation of tissue homeostasis resulting in organ growth based on a dysbalance between cell replication and cell death by apoptosis [7,8]. This dysbalance acts also on mutated/initiated cells. By this mechanism NGCs enhance the selective proliferation of preneoplastic cells and exert tumour promoting effects. Possible molecular mechanisms of the tumour promoting effects of NGCs comprise epigenetic changes such as hypo- and hypermethylation of CpG sites, chromatin modifications, and miRNA regulated mechanisms [9], but also endocrine effects, inhibition of gap junctional intercellular communications, immune modulation, and/or profound disturbances in the epithelial-mesenchymal interactions [6]. It is known that during carcinogenesis the microenvironment may gain a pivotal role supporting preneoplastic and neoplastic cell growth via an altered vasculature, deviated immunological activities and altered interstitial extracellular matrix (ECM) [10,11]. Further possible modes of NGC actions in rodents may be cytotoxicity followed by regenerative growth and a pro-inflammatory status. Involvement of inflammatory mechanisms may be accompanied by enhanced production of reactive oxygen species (ROS) and reactive nitrogen species (RNS) [7,8]. These two species may have relevance in carcinogenesis via signalling function and possibly cause endogenous DNA damage, which may be responsible for a weak genotoxic potential of NGCs.

The most common target organ of NGCs in rodent models is the liver, i.e., about 40% of all NGC tested so far are hepatocarcinogens. Hitherto, research on the action of NGCs has been focusing mainly on hepatocytes (HCs), the major parenchymal cells of the liver which eventually give rise to liver cancer. A role of non-parenchymal liver cells (NPCs) in NGC-driven hepatocarcinogenesis has been mostly neglected, because these cells do not transform [12,13]. However, NPCs, which consists mainly of Kupffer, endothelial, and stellate cells, may also be targeted by NGCs and may contribute considerably to the selective proliferation of preneoplastic and neoplastic HCs via release of paracrine growth factors or other growth-enhancing stimuli. Here, as a model NGC we chose phenobarbital (PB), a barbiturate known to be a potent tumour promoter in rodent liver [14–18]. PB has been described to interact with the pregnane X receptor (PXR) and the constitutive androstane receptor (CAR) triggering a signal transduction cascade leading to an induction of cytochrome P450 genes such as members of the CYP2B and CYP3A subfamily [9,19–23]. Furthermore, it was shown that PB acts through other mechanisms such as oxidative stress [24], which may correlate with the P450 induction [25], driving tumour promotion by inducing proliferation [26,27] in HCs, non-receptor mediated endocrine modifications and inhibition of gap junction intercellular communications, regulating growth and differentiation [18]. 

Proteome profiling is a powerful technique to observe molecular consequences of drug action. Cells may respond to drug actions via the synthesis of new proteins. Cells synthesize proteins in order to overcome biological challenges. Therefore, the identification of drug-induced proteins may give important hints to better understand the way of action of drugs. Furthermore, if the drug-induced proteins display restricted expression patterns, they may be used as indicative marker proteins. For comprehensive investigation, we analysed subcellular fractions including the secretome of isolated HCs and NPCs separately applying LC-MS/MS analysis. We hypothesized that NGC-driven hepatocarcinogenesis may involve NPCs and specifically considered a potential contribution of inflammatory activities of this tissue compartment, as known for the pathogenesis of hepatocellular carcinoma in humans [28]. Therefore we treated HCs and NPCs with pro-inflammatory cytokines *in vitro* in order to identify proteome signatures characteristic for such events. Induction of such a signature by PB treatment could thus indicate the involvement of inflammatory processes in drug action. Furthermore, we investigated PB effects induced by *in vivo* treatment of animals in comparison to PB effects observed upon *in vitro* treatment of isolated primary cells, including both HCs and NPCs. This strategy provided the unique opportunity to differentiate direct drug effects on the isolated cell types *in vitro* from indirect drug effects modulated by complex epithelial-mesenchymal interactions in the intact organism. This approach may thus give novel insights into the mode of action of this prototypic NGC. To conclude, the aim of the present study was to investigate proteome alterations in HCs as well as NPCs, which are caused by the non-genotoxic carcinogen phenobarbital (PB) *in vitro* as well as *in vivo*.

## Material and Methods

### Animals and treatment

Male Wistar rats were obtained from and kept at the ‘‘Division for Decentralized Biomedical Facilities of the Medical University of Vienna” under standardized SPF-conditions. Phenobarbital (PB, 5-Ethyl-5-phenyl-barbituric acid-sodium salt; Fluka, 04712) [29,30], admixed to drinking water, was administered to three rats which were 8 to 10 weeks old at a daily dose of 50mg/kg bodyweight. Two times a week the PB concentrations in the drinking water were adjusted to the body weights and the amount of water consumed. Five controls and four animals for the *in vitro* experiments were treated with tap water only. Animals were sacrificed by exsanguination under CO_2_-asphyxation during the liver perfusion. The experiments were approved by the ‘‘Committee of Animal Protection of the Austrian Ministry of Sciences” (permission number 66009/157 II10b/2009) and performed according to Austrian regulations in accordance with the criteria outlined in the ‘‘Guide for the Care and Use of Laboratory Animals” by the National Academy of Sciences.

### Separation of liver cells and primary cultures

The following procedure was used for the *in vitro* and the *in vivo* experiments. To isolate and cultivate hepatocytes (HCs) and non-parenchymal cells (NPCs) at a functional state as naïve as possible, livers of rats were perfused with collagenase (Worthington CLS-2) as described before [31,32]. In the resulting cell suspension HCs were isolated from NPCs by low speed centrifugation followed by discontinuous density gradient centrifugation using Percoll [33]. The purity was found to be an average of 95.4% for hepatocyte fractions and 99.8% for NPC fractions. Cell preparations were used for further experimentation when the viability exceeded 90%, as determined by the trypan blue exclusion assay. HCs and NPCs were subsequently seeded on collagen-coated 6-well plates. HCs were seeded at a density of 4x10^5^cells/well in Williams’ medium E (Invitrogen) supplemented with glutamax, HEPES, gentamycin, H2 mix, ascorbat and 10% FCS. NPCs were seeded at a density of 3-4x10^6^cells/well in RPMI 1640 medium (Gibco Ltd.) supplemented with gentamycin and 10% FCS at 37°C for 2 hours. To determine the purity of the isolated cell fractions, cells were counted using microscope pictures and the ImageJ software (National Institutes of Health). After an attachment period of 2 hours cells were switched to serum-free medium (Williams medium E and RPMI 1640 medium, both supplemented as above without the 10% FCS) and kept at 37°C for further 24 hours in order to collect cell supernatants. 

### In vitro treatment of primary HCs and NPCs

In addition to the procedure described above, the *in vitro* treatment of cultures commenced 2h after plating of cells deriving from four untreated rats. Two rats were used for the cytokine treatment, while the other two were used for the PB treatment. HCs were treated with 10ng/ml interleukin-1ß (R&D Systems) and 5ng/ml interleukin-6 (R&D Systems) [34] for 24 hours, which induce the acute phase plasma protein synthesis in HCs [35–38]. 10ng/milliliter lipopolysaccharide (LPS, Sigma-Aldrich) was applied to the medium of NPCs for 24 hours [39–43]. HCs and NPCs were treated with 1mM PB (Fluka) for 24 hours.

### Cellular sub-fractionation and protein sample processing (see also 44)

The serum-free supernatants were sterile filtered using a 0.2µm cellulose acetate filter (Whatman). One part of this filtrate was precipitated by adding 5x volume of -20°C tempered p.a. ethanol (Merck) and subsequent storing at -20°C for at least overnight. The other part was directly stored at -80°C for subsequent analyses by ELISA. During all steps samples were kept on ice. For harvesting of cytoplasmic and nucleic protein fraction, cells were gathered in isotonic buffer (10mM HEPES/NaOH pH=7.4, 10mM NaCl, 3.5mM MgCl2, 1mM EGTA, 0.25M Sucrose and 0.5% Triton X-100) and protease inhibitor mix (1mM PMSF; aprotinin, leupeptin and pepstatin, [1µg/ml] each). The cells were disrupted by sheer stress caused by syringing the cell lysates through 23G needles. The cytoplasmic fraction, in the supernatant, was separated from nuclei and membrane proteins as well as debris by centrifugation at 2300xg and 4°C for 5min and was subsequently precipitated by ethanol tempered to -20°C. The nuclei protein fraction was extracted from the remaining residue via a 10min incubation with an extraction buffer (10mM Tris/HCl pH=7.4, 1mM EDTA, 500mM NaCl) which act through osmotic pressure followed by a 1:10 dilution with a NP40 buffer (10mM Tris/HCl pH=7.4, 1mM EDTA, 0.5% NP40), to reduce the final NaCl concentration, for 15min. Nucleic proteins were separated from debris by centrifugation at 2328xg and 4°C for 5min and precipitated by ethanol tempered to -20°C.

After precipitation all fractions were centrifuged at 4700xg and 4°C for 25min. The resulting protein pellets were dissolved in sample buffer (7.5M urea, 1.5M thiourea, 4% CHAPS, 0.05% SDS, 100mM DTT) in a volume according to the protein amount / pellet size. 

### Shotgun analysis - 1D-SDS PAGE, in-gel tryptic digestion & MS analysis ('bottom up')

For 1D-SDS PAGE we used a 4% stacking and a 12% resolving polyacrylamide gel. Protein samples were loaded onto the gel and the electrophoresis ran until complete separation of a pre-stained molecular marker (Dual Color, Biorad, Hercules, CA) was visible. Gels were fixated with 50% methanol/10% acetic acid and MS compatible silver stained as described by Mortz, E. *et al* [45]. For tryptic digestion samples were cut into lanes to group proteins with a similar molecular weight, which improved the following LC-MS/MS analyses. These lanes were cubed and the proteins were destained, reduced and alkylated before digestion with trypsin (sequencing grad, Roche) at 37°C over night as described before [46]. After elution the peptide solutions were analysed by LC - MS/MS.

For reversed-phase chromatography we used a nano-flow LC (1100 Series LC system, Agilent) combined with the HPLC chip technology (Agilent). The chips consist of a 40nl Zorbax 300SB-C18 trapping column and a Zorbax 300SB-C18 (75µm x 150mm) separation column. The flow rate was 400nl/min, using a gradient from 0.2% formic acid and 3% ACN to 0.2% formic acid and 50% ACN over 40min or 60min for supernatants or the other two fractions, respectively.

Peptide identification was performed by MS/MS fragmentation analysis using an ion-trap mass spectrometer (XCT-Ultra, Agilent) combined with already described HPLC chips, which are eventually an orthogonal nanospray ion source (Agilent). For peak list-generation and spectrum identification, of the MS/MS data we used Spectrum Mill MS Proteomics Workbench software (Version A.03.03, Agilent). We searched our MS/MS data against the UniProtKB/Swiss-Prot protein database (Version 10^th^ August 2010; 519,348 entries). The settings were as follows: max. of 2 missed cleavages, minimum scored peak intensity (%SPI) of 70%, precursor mass tolerance of +/-1.5Da, product mass tolerance +/- 0.7. Two types of modification were considered, both arising during sample preparation. Carbamidomethylation of cysteine (deliberately) was set as fixed modification and oxidized methionine (artefact) was set as post-translational modification.

### Generating protein maps and their interpretation

The resulting peptide-protein assignment list was reviewed by hand considering the following parameters. Spectrum Mill peptide sequence score values (sequence matching probability) of >13 counted as valid sequence assignments. Peptide sequences were valid, when at least one peptide sequence scored as much and did not occur in other proteins of this cell type. An estimated error rate was calculated by searching the sequences against a non-sense reversed database. Furthermore we included peptides scoring between 9 and 13 only if precursor m/z value, retention time and MS2 pattern were found similarly in at least one of our previous experiments and the peptide was thereby scoring above 13. With respect to protein inference, we chose the smallest number of proteins required to explain all observed peptides as described for ProteinProphet [47]. As our protein identification algorithm includes manual selection, we cannot calculate an exact false discovery rate. 

Proteome analysis using emPAI (exponentially modified protein abundance index) values[48], comparisons and PB specific alterations were recorded and interpreted using the GPDE (Griss Proteomics Database Engine), a database specifically engineered for the identification and characterization of marker proteins [49]. 

### ELISA

Arginase-1 protein levels were determined by a kit (Uscn Life Science Inc.; Houston, TX 77036) according to the user manual.

## Results

### Isolation and proteome profiling of primary rat hepatocytes and non-parenchymal cells

**Figure 1:**
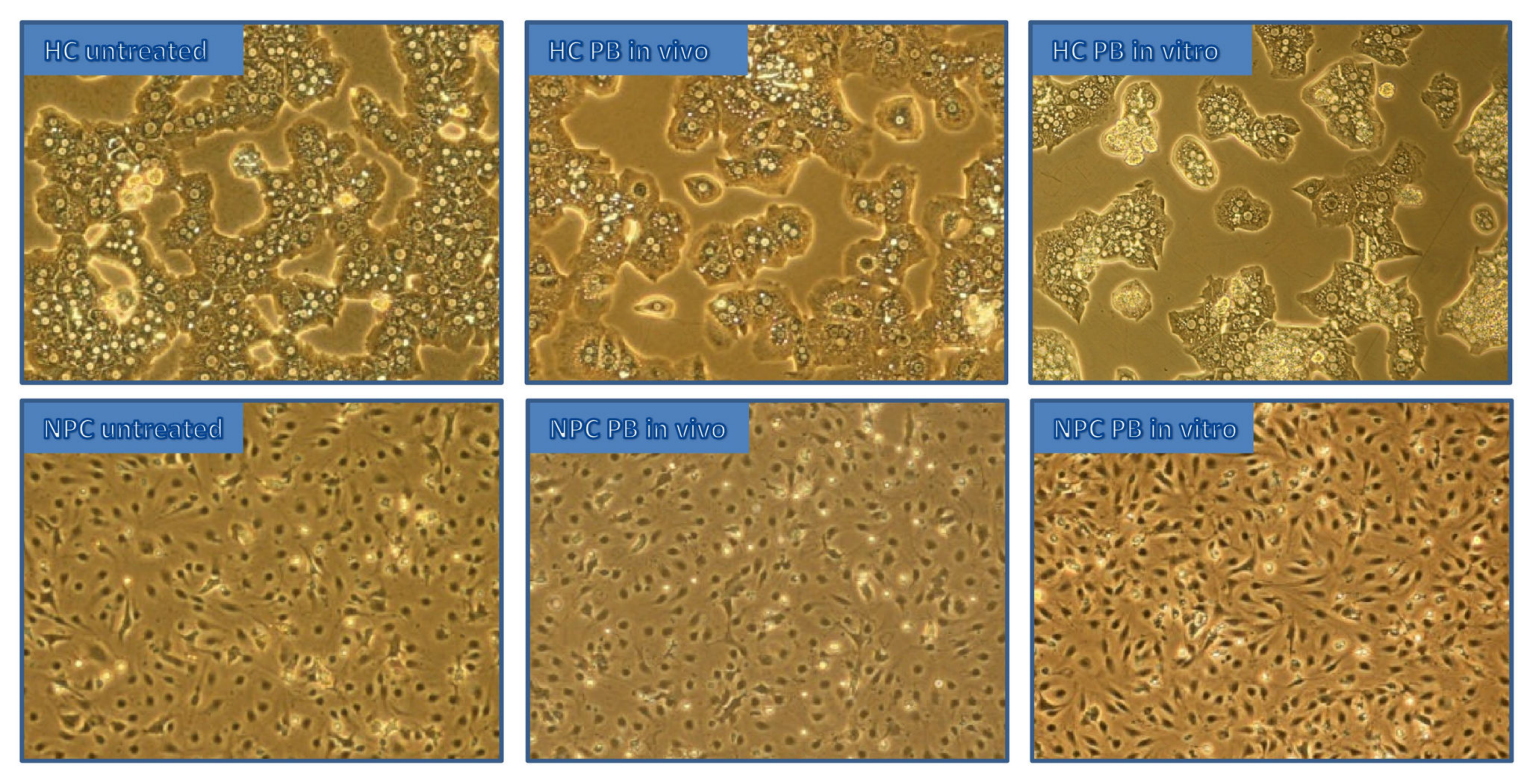
Microscope images of HCs and NPCs in culture. These pictures depict representative areas of untreated, PB *in* *vitro* as well as *in* *vivo* treated HCs and NPCs in culture extracted from microscopic images of equal magnification.

Primary rat cells were obtained from untreated rats and rats treated with PB *in vivo*. Cell isolation was performed by liver perfusion followed by separation into hepatocytes (HCs) and non-parenchymal cells (NPCs) (see Figure *1*). Cells were cultured for 24 hours to allow accumulation of secreted proteins. Subsequently, cells were fractionated into cytoplasm and nuclear extract. The protein fractions were further separated by SDS-PAGE and forwarded to proteome profiling via LC-MS/MS as described.

We restricted our analyses to proteins which were identified by two peptides or more in two or more independent experiments. By this approach, we identified 1148 proteins in HCs and 1213 proteins in NPCs with an overlap of 966 proteins in both cell compartments. The 182 proteins being apparently specific for HCs indeed comprise a large number of known liver-specific proteins including, apolipoproteins and other serum proteins, cytochrome P450 isoenzymes, sulfotransferases, UDP-glucuronosyltransferases and many others (table *1*-*5*, table *S1* & *S2*). Furthermore, the 247 proteins identified in NPCs included many proteins known to be expressed in stromal cells. Representative for leukocytes are the surface antigens CD37, CD47, CD96 and CD166 as well as the chemokines CXCL1, CXCL2, and CCL6. Amongst known marker proteins for endothelial cells we identified endothelial cell-specific molecule 1, endothelial nitric oxide synthase and septin-2 (vascular endothelial cell specific protein 11), while MMP-3 and various collagens are characteristic for the stellate cells.

**Table 1:** Selected proteins found up-regulated in the secretome of HCs and NPCs isolated from rat livers, when treated with PB *in vivo.*

**Hepatocytes / secreted protein fraction**		
**Accession number**	**Protein name**	**GO - biological process**
P24090	Alpha-2-HS-glycoprotein	acute-phase response
P06238 ^t^	Alpha-2-macroglobulin	acute-phase response, response to glucocorticoid stimulus
P02650	Apolipoprotein E	cellular response to growth factor stimulus
P02454	Collagen alpha-1(I) chain	cellular response to transforming growth factor beta stimulus, response to corticosteroid stimulus
P06759 ^t^	Apolipoprotein C-III	inflammatory & drug response
P02680	Fibrinogen gamma chain	inflammatory response
**Non-parenchymal cells / secreted protein fraction**		
**Accession number**	**Protein name**	**GO - biological process**
P29534 ^t^	Vascular cell adhesion protein 1 (V-CAM 1)	acute & chronic inflammatory response
P12346	Serotransferrin (Transferrin) (Beta-1 metal-binding globulin) (Liver regeneration-related protein LRRG03)	acute-phase response
P07154	Cathepsin L1 (Major excreted protein) (MEP) (Cyclic protein 2) (CP-2)	autophagic cell death, proteolysis, response to organic cyclic compound
P22985	Xanthine dehydrogenase/oxidase	bone resorption, contributes to the generation of reactive oxygen species.
P11232	Thioredoxin (Trx)	cellular response to drug
P02761	Major urinary protein (MUP) (Alpha-2u-globulin)	cellular response to lipid, positive regulation of gene expression
P10760	Adenosylhomocysteinase (AdoHcyase) (S-adenosyl-L-homocysteine hydrolase)	chronic inflammatory response to antigenic stimulus
P07152 ^t^	Stromelysin-2 (SL-2) (Matrix metalloproteinase-10) (MMP-10)	Collagen degradation
P18484	AP-2 complex subunit alpha-2 (Alpha2-adaptin)	endocytosis, intracellular protein transport
P15978	Class I histocompatibility antigen, Non-RT1.A alpha-1 chain	immune response
Q63228	Glia maturation factor beta (GMF-beta)	inhibition of proliferation of tumor cells
P31720	Complement C1q subcomponent subunit A	innate immune response
Q711G3 ^t^	Isoamyl acetate-hydrolyzing esterase 1 homolog	lipid catabolic process
P14841	Cystatin-C (Cystatin-3)	positive regulation of cell proliferation, response to drug and toxin
P20961 ^t^	Plasminogen activator inhibitor 1 (PAI-1) (Endothelial plasminogen activator inhibitor) (Serpin E1)	response to reactive oxygen species, tissue regeneration

These up-regulated proteins of interest were selected out of 666 and 1044 distinct proteins, whereby the selected proteins had a relative difference of emPAI values of at least 40% and 50% for HCs and NPCs, respectively. Furthermore, the proteins had to fulfill the criteria of being represented in at least 50% of the experiments with at least 2 peptides. For convenience, the proteins are sorted according their Go terms.

t Evidence at transcript level only

**Table 2:** Selected proteins found up-regulated in the cytoplasmic protein fraction of HCs and NPCs isolated from rat livers, when treated with PB *in* *vivo*.

**Hepatocytes / cytoplasmic protein fraction**			
**Accession number**	**Protein name**		**GO - biological process**
P09034 ^t^	Argininosuccinate synthase		acute-phase response, cellular response to interferon-gamma, response to drug, liver development
P12346	Serotransferrin		acute-phase response, response to organic cyclic compound
Q6P791	Regulator complex protein LAMTOR1		cell growth, cholesterol homeostasis, positive regulation of MAPK & TOR signaling cascade
Q8K581 ^t^	Thioredoxin domain-containing protein 9		cell redox homeostasis
P15709 ^t^	Bile salt sulfotransferase		drug metabolic process
P00176	Cytochrome P450 2B1		drug metabolic process
P05178 ^t^	Cytochrome P450 2C6		drug metabolic process
P04903 ^t^	Glutathione S-transferase alpha-2		drug metabolic process
P17988	Sulfotransferase 1A1		drug metabolic process
P08011	Microsomal glutathione S-transferase 1		drug metabolic process
P09875 ^t, PB^	UDP-glucuronosyltransferase 2B1		drug metabolic process
P19488 ^t, PB^	UDP-glucuronosyltransferase 2B37		drug metabolic process
P97675	Ectonucleotide pyrophosphatase / phosphodiesterase family member 3 (E-NPP 3) (B10)		immune response
P07151	Beta-2-microglobulin		immune response, antigen processing and presentation of peptide antigen via MHC class I
P80254	D-dopachrome decarboxylase		inflammatory response
P51647	Retinal dehydrogenase 1		response to oxidative stress, response to organic cyclic compound
P55053	Fatty acid-binding protein, epidermal		response to wounding
Q66HA8	Heat shock protein 105 kDa		stress response
**Non-parenchymal cells / cytoplasmic protein fraction**			
**Accession number**		**Protein name**	**GO - biological process**
P11497 ^PB^		Acetyl-CoA carboxylase 1	acetyl-CoA metabolic process, fatty acid biosynthetic process, response to organic cyclic compound
Q4KM33 ^t, PB^		Pleckstrin	actin cytoskeleton reorganization, hemopoietic progenitor cell differentiation, positive regulation of platelet activation
P12346		Serotransferrin	acute-phase response, response to organic cyclic compound
Q70VB1 ^PB^		G-protein coupled receptor family C group 6 member A	calcium-mediated signaling, response to amino acid stimulus
P85972		Vinculin	cell adhesion
Q91Y81		Septin-2 (Vascular endothelial cell specific protein 11)	cell division
Q9ESH6 ^t^		Glutaredoxin-1	cell redox homeostasis
P85845		Fascin	cellular response to cell-matrix adhesion, liver development, cell motility
Q2PQA9 ^t, PB^		Kinesin-1 heavy chain	cytoplasm organization, vesicle transport along microtubule
P00176 ^PB^		Cytochrome P450 2B1	drug metabolic process, response to organic cyclic compound
P07323 ^PB^		Gamma-enolase	glycolysis, gluconeogenesis, response to organic cyclic compound
P97675 ^PB^		Ectonucleotide pyrophosphatase/phosphodiesterase family member 3	immune response
P67779		Prohibitin	organ regeneration, response to cytokine stimulus, response to drug, response to stress
Q64244 ^t, PB^		ADP-ribosyl cyclase 1	positive regulation of B cell proliferation, cell growth & vasoconstriction and response to drug
Q99J82 ^t^		Integrin-linked protein kinase (cell-cell junction)	positive regulation of MAPKKK cascade, positive regulation of cell migration, positive regulation of cell proliferation
Q5U204 ^t^		Ragulator complex protein LAMTOR3	positive regulation of TOR signaling cascade, cellular protein localization
P13832 ^t, PB^		Myosin regulatory light chain RLC-A	protein targeting to plasma membrane, regulation of cell shape
Q62950 ^PB^		Dihydropyrimidinase-related protein 1	pyrimidine base catabolic process
Q5FVC7 ^t, PB^		Arf-GAP with coiled-coil, ANK repeat and PH domain-containing protein 2	regulation of ARF GTPase activity
P05943 ^PB^		Protein S100-A10	regulation of cell differentiation, regulation of cell growth
P25235 ^t^		Dolichyl-diphosphooligosaccharide--protein glycosyltransferase subunit 2	response to drug
P20961 ^t^		Plasminogen activator inhibitor 1 (PAI-1)	response to reactive oxygen species, tissue regeneration, positive regulation of receptor-mediated endocytosis
O88600		Heat shock 70 kDa protein 4	stress response
Q7TP47 ^t^		Heterogeneous nuclear ribonucleoprotein Q (hnRNP Q) (Liver regeneration-related protein LRRG077)	tissue regeneration, mRNA processing
Q68FP1		Gelsolin (Actin-depolymerizing factor)	tissue regeneration, regulation of cell adhesion
P50399		Rab GDP dissociation inhibitor beta	vesicle-mediated protein transport
Q5U2R7 ^t, PB^		LDLR chaperone MESD	Wnt receptor signaling pathway

These up-regulated proteins of interest were selected out of 1283 and 1336 distinct proteins, whereby the selected proteins had a relative difference of emPAI values of at least 50% for HCs and NPCs, with the same exclusion criteria as table 1. For convenience, the proteins are sorted according their Go terms.

t Evidence at transcript level only, ^PB^ – found only in PB treated rats

**Table 3:** Selected proteins found up-regulated in the nuclear extract protein fraction of HCs and NPCs isolated from rat livers, when treated with PB *in* *vivo*.

**Hepatocytes / nuclear extract protein fraction**		
**Accession number**	**Protein name**	**GO - biological process**
P28064	Proteasome subunit beta type-8 (Proteasome subunit beta-5i)	anti-gen processing and presenting, fat cell differentiation
P13383	Nucleolin (Protein C23)	associated with transcription
Q6TRW4 ^t, PB^	Sister chromatid cohesion protein PDS5 homolog B	cell division
Q5I0H9 ^t^	Protein disulfide-isomerase A5	cell redox homeostasis, response to stress
Q9WUL0 ^t^	DNA topoisomerase 1	cellular response to stress
P27008 ^PB^	Poly [ADP-ribose] polymerase 1	DNA damage response, detection of DNA damage
P05183 ^PB^	Cytochrome P450 3A2	drug metabolic process, oxidative demethylation
O09171	Betaine--homocysteine S-methyltransferase 1	methionine biosynthetic process, protein methylation
Q4KM65 ^t^	Cleavage and polyadenylation specificity factor subunit 5	mRNA polyadenylation
Q62780	Probable ATP-dependent RNA helicase DDX46	mRNA processing
P17136 ^t^	Small nuclear ribonucleoprotein-associated protein B (snRNP-B)	mRNA processing
O35821 ^t^	Myb-binding protein 1A (PAR-interacting protein) (PIP)	nucleocytoplasmic transport, transcription (DNA-dependent)
Q6LED0	Histone H3.1	nucleosome assembly
Q00715	Histone H2B type 1*	nucleosome assembly
Q6P747 ^t, PB^	Heterochromatin protein 1-binding protein 3	nucleosome assembly
P62914	60S ribosomal protein L11	protein localization to nucleus, translation
P07895	Superoxide dismutase [Mn], mitochondrial	removal of superoxide radicals
Q6AYB5 ^t, PB^	Signal recognition particle 54 kDa protein	SRP-dependent cotranslational protein targeting to membrane
Q6P7R8 ^t, PB^	Estradiol 17-beta-dehydrogenase 12	steroid biosynthetic process
Q63396 ^t, PB^	Activated RNA polymerase II transcriptional coactivator p15	transcription, DNA-dependent
Q6PDV7	60S ribosomal protein L10	translation
P05765	40S ribosomal protein S21	translation
P24050 ^t^	40S ribosomal protein S5	translation
Q71TY3 ^t^	40S ribosomal protein S27	translation
P43244	Matrin-3 (Nuclear scaffold protein p130/MAT3)	chromatin organisation
**Non-parenchymal cells / nuclear extract protein fraction**		
**Accession number**	**Protein name**	**GO - biological process**
P41516 ^t^	DNA topoisomerase 2-alpha	DNA topological change, response to drug
O08629	Transcription intermediary factor 1-beta (TIF1-beta) (epithelial to mesenchymal transition)	epithelial to mesenchymal transition, positive regulation of transcription (DNA-dependent)
Q68FY1	Nucleoporin NUP53	mRNA & protein transport
Q6AY87 ^t^	THO complex subunit 6 homolog (WD repeat-containing protein 58)	mRNA processing
Q4V898	RNA-binding motif protein, X chromosome	mRNA splice site selection, positive regulation of transcription (DNA-dependent)
Q00566	Methyl-CpG-binding protein 2 (MeCp-2 protein)	negative regulation of transcription from RNA polymerase II promoter, transcription (DNA-dependent)
Q9Z2Y1	Protein timeless homolog (rTIM)	positive regulation of circadian rhythm, response to DNA damage stimulus
Q9JIL3	Interleukin enhancer-binding factor 3	protein methylation, transcription (DNA-dependent)

These up-regulated proteins of interest were selected out of 1081 and 957 distinct proteins, whereby the selected proteins had a relative difference of emPAI values of at least 50% for HCs and NPCs, with the same exclusion criteria as table 1. For convenience, the proteins are sorted according their Go terms.

* Exact isoform could not be distinguished with our resources

t Evidence at transcript level only, ^PB^ – found only in PB treated rats

**Table 4:** Selected proteins found down-regulated in HCs isolated from rat livers, when treated with PB *in* *vivo*.

**Accession number**	**Protein name**	**GO - biological process**
**Secreted protein fraction**		
Q2TL32 ^t^	E3 ubiquitin-protein ligase UBR4 (N-recognin-4) (Zinc finger UBR1-type protein 1)	Ubl conjugation pathway; together with clathrin, forms meshwork structures involved in membrane morphogenesis and cytoskeletal organization
**Cytoplasmic protein fraction**		
P08516	Cytochrome P450 4A10 (CYPIVA10) (Cytochrome P450-LA-omega 1) (Cytochrome P452)	arachidonic acid metabolic process
P09606	Glutamine synthetase (GS) (Glutamate--ammonia ligase) (Glutamate decarboxylase)	ammonia assimilation cycle, glutamine biosynthetic process
P04182	Ornithine aminotransferase, mitochondrial (Ornithine--oxo-acid aminotransferase)	L-proline biosynthetic process
Q5PPL3 ^t^	Sterol-4-alpha-carboxylate 3-dehydrogenase, decarboxylating	Cholesterol biosynthesis, Steroid biosynthesis
**Nuclear extract protein fraction**		
Q9ES53	Ubiquitin fusion degradation protein 1 homolog (UB fusion protein 1)	proteasomal ubiquitin-dependent protein catabolic process

These down-regulated proteins of interest were selected out of 389 proteins. These proteins were found exclusively in the livers of untreated rats and had to fulfill the criteria of being represented in at least 50% of the experiments with at least 2 peptides.

t Evidence at transcript level only,

**Table 5:** Selected proteins found down-regulated in NPCs isolated from rat livers, when treated with PB *in* *vivo*.

**Accession number**	**Protein name**	**GO - biological process**
**Secreted protein fraction**		
Q5XI22	Acetyl-CoA acetyltransferase, cytosolic (Cytosolic acetoacetyl-CoA thiolase)	liver development, cellular response to nutrient
P52844 ^t^	Estrogen sulfotransferase, isoform 1 (EST-1)	estrogen metabolic process
P09606	Glutamine synthetase (GS) (Glutamate--ammonia ligase) (Glutamate decarboxylase)	ammonia assimilation cycle, glutamine biosynthetic process
P14095	Growth-regulated alpha protein (C-X-C motif chemokine 1) (Cytokine-induced neutrophil chemoattractant 1) (CINC-1)	acute inflammatory response, immune response
P10868	Guanidinoacetate N-methyltransferase	S-adenosylhomocysteine metabolic process
P04176	Phenylalanine-4-hydroxylase (PAH) (Phe-4-monooxygenase)	L-phenylalanine metabolic process
**Cytoplasmic protein fraction**		
Q5DWV2 ^t^	Cadherin-7	homophilic cell adhesion
O35796	Complement component 1 Q subcomponent-binding protein, mitochondrial (Glycoprotein gC1qBP)	negative regulation of interferon-gamma & interleukin-12 production, positive regulation of apoptotic process
Q64611	Cysteine sulfinic acid decarboxylase (Sulfinoalanine decarboxylase)	carboxylic acid metabolic process
P10818	Cytochrome c oxidase subunit 6A1, mitochondrial (Cytochrome c oxidase polypeptide VIa-liver)	mitochondrial respiratory chain complex IV
P08683	Cytochrome P450 2C11 (CYPIIC11) (Cytochrome P-450(M-1))	xenobiotic metabolic process
P63095	Guanine nucleotide-binding protein G(s) subunit alpha isoforms short	adenylate cyclase-activating dopamine receptor signaling pathway
P14659 ^t^	Heat shock-related 70 kDa protein 2 (Heat shock protein 70.2)	multicellular organismal development, response to stress
P27881	Hexokinase-2 (Hexokinase type II) (HK II)	cellular glucose homeostasis, apoptotic mitochondrial changes
Q920F3	KH domain-containing, RNA-binding, signal transduction-associated protein 2 (SLM-1)	regulation of transcription, DNA-dependent
O08816	Neural Wiskott-Aldrich syndrome protein (N-WASP)	actin polymerization or depolymerization
P04182	Ornithine aminotransferase, mitochondrial (Ornithine--oxo-acid aminotransferase)	L-proline biosynthetic process
P22062	Protein-L-isoaspartate(D-aspartate) O-methyltransferase (PIMT)	S-adenosylhomocysteine metabolic process
P12928 ^t^	Pyruvate kinase isozymes R/L (L-PK)	ATP biosynthetic process
Q53B90 ^t^	Ras-related protein Rab-43	protein transport
Q6BBI8 ^t^	Ubiquitin-fold modifier-conjugating enzyme 1 (Ufm1-conjugating enzyme 1)	protein ufmylation
**Nuclear extract protein fraction**		
P21531	60S ribosomal protein L3 (L4)	translation
P08753	Guanine nucleotide-binding protein G(k) subunit alpha (G(i) alpha-3)	cell cycle, adenylate cyclase-inhibiting G-protein coupled receptor signaling pathway
P63095	Guanine nucleotide-binding protein G(s) subunit alpha isoforms short (G-alpha-8)	adenylate cyclase-activating dopamine receptor signaling pathway, heterotrimeric G-protein complex
P14659 ^t^	Heat shock-related 70 kDa protein 2 (Heat shock protein 70.2)	multicellular organismal development, response to stress
P17955	Nuclear pore glycoprotein p62 (62 kDa nucleoporin) (Nucleoporin Nup62)	cell death, negative regulation of cell proliferation
P62961	Nuclease-sensitive element-binding protein 1 (CCAAT-binding transcription factor I subunit A) (DNA-binding protein B) (EFI-A) (YB-1)	CRD-mediated mRNA stabilization
Q498U4	SAP domain-containing ribonucleoprotein (Nuclear protein Hcc-1)	regulation of transcription, DNA-dependent

These down-regulated proteins of interest were selected out of 484 proteins. These proteins were found exclusively in the livers of untreated rats and had to fulfill the criteria of being represented in at least 50% of the experiments with at least 2 peptides.

t Evidence at transcript level only,

### In vitro treatment of isolated primary cells

**Figure 2:**
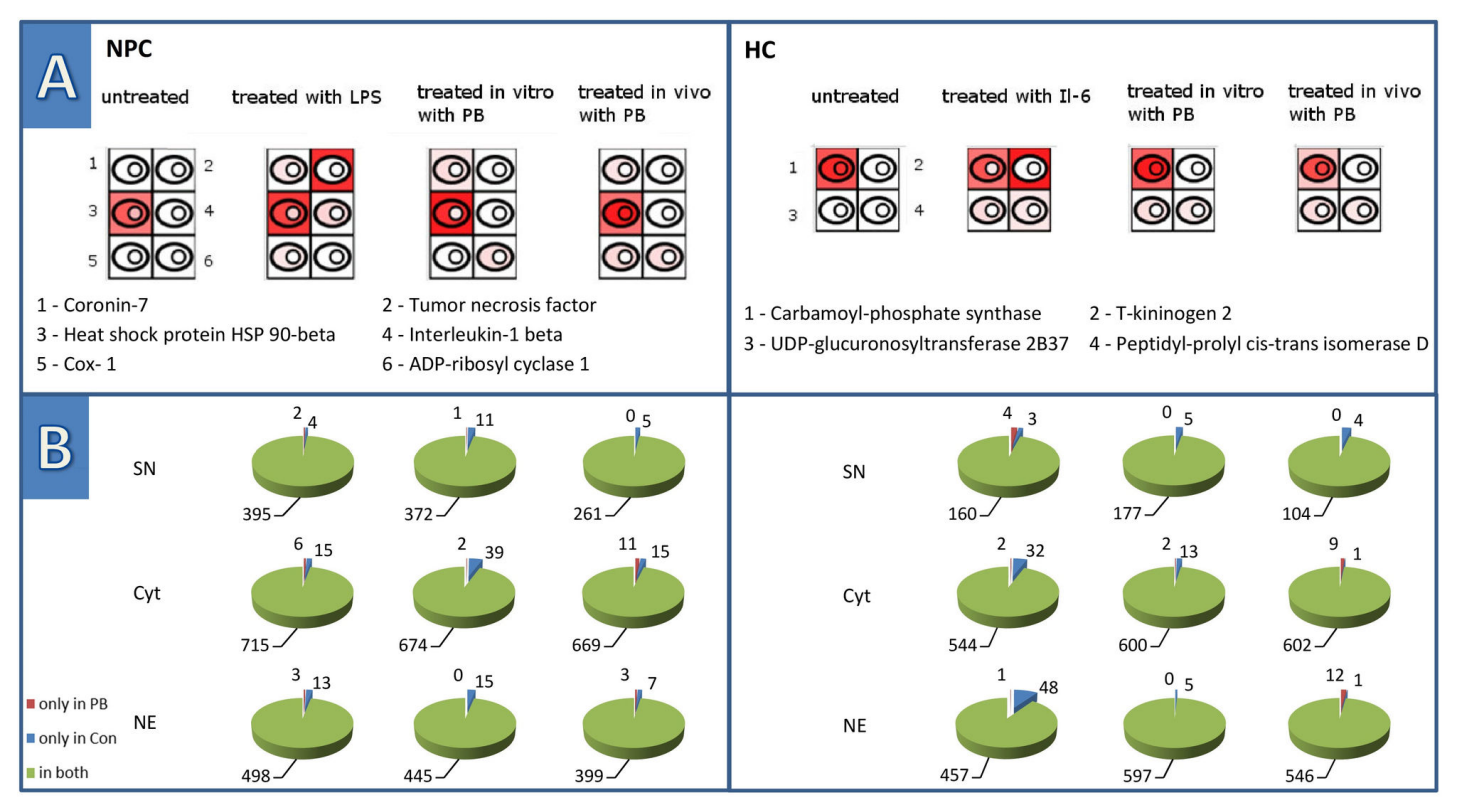
Proteome alterations induced by *in* *vitro* treatment of primary cells. Part A) shows schematic representations of a cell and her three sub-compartments, namely the supernatant, the cytoplasm and the nucleus. The intensity of red represents the degree of amount of the selected protein found in the respective compartment in contrast to the other experiments. The higher intensity of red corresponds to a higher occurrence. This allows an easy comparison of the expression levels of a protein in different experimental setups. NPCs induce the secretion of IL-1beta and TNF-alpha upon inflammatory stimulation with LPS. *In* *vitro* treatment with PB induced coronin-7 and ADP-ribosyl cyclase 1, which both are also induced by *in* *vivo* treatment. The expression of Hsp90, a stress response related protein, was increased upon LPS and PB treatment. Prostaglandin, a protein involved in promotion of proliferation in normal and preneoplastic cells, was induced upon LPS and in vivo PB treatment. HCs respond hardly to the *in* *vitro* treatment with PB. Treatment with IL-6 specifically induced the acute phase protein T-kininogen-2. UDP-glucuronosyltransferase 2B37 and the chaperone peptidyl-prolyl cis-trans isomerase D were induced by both *in* *vitro* stimulation experiments as well as by the *in* *vivo* treatment with PB. Carbamoyl-phosphate synthase is part of the urea cycle and has to be found in all four categories. Proteins in NPC: (1) **O35828** Coronin-7, (2) **P16599** Tumor necrosis factor, (3) **P34058** Heat shock protein HSP 90-beta, (4) **Q63264** Interleukin-1 beta, (5) **Q63921** Prostaglandin G/H synthase 1, (6) **Q64244** ADP-ribosyl cyclase 1. Proteins in HC: (1) **P07756** Carbamoyl-phosphate synthase [ammonia], (2) **P08932** T-kininogen 2, (3) **P19488** UDP-glucuronosyltransferase 2B37, (4) **Q6DGG0** Peptidyl-prolyl cis-trans isomerase D. Part B) demonstrates the distribution of distinct proteins within the three fractions, supernatant, cytoplasm and nuclear protein fractions, underneath the respective treatment of the cells, which gives an overview of the responsiveness of the cells. Abbr.: SN –proteome of the supernatant, Cyt – proteome of the cytoplasm, NE – proteome of the nuclear extract.

HCs were treated with IL-6 and NPCs with LPS, respectively. Upon treatment for 24 hours, the induction of several pro-inflammatory proteins was observed which confirms the responsiveness of these cells to stress stimuli under our experimental conditions (Figure *2*). 

To investigate direct drug effects, primary untreated cells were treated with 1mM PB for 24 hours and analysed by proteome profiling. Remarkably, this treatment had hardly any measureable effect on the proteome composition of HC and NPCs. A single protein was apparently induced in HC and two proteins only in NPCs, respectively. Proteins indicating stress such as chaperones including the heat shock protein family, stress-induced phosphoprotein, heme oxygenase 1 and DNAJ homolog subfamily members were not or hardly induced. 

### In vivo treatment of animals with phenobarbital

13 proteins were found to be newly induced in the cytoplasmic fraction of HCs by PB, including phase I drug metabolizing enzymes such as amine oxidase, phase II enzymes such as UDP-glucuronosyltransferases and glutathione S-transferases, the chaperone rotamase D, glutathione synthetases and the proto-oncogen c-Raf. The induction of estradiol 17-beta-dehydrogenase 8 may indicate alterations in steroid hormone homeostasis. The induction of CYP2B, probably via CAR, is a known positive control for PB action.

Table *1*, *2* and *3* depict the most significant proteins found up-regulated in the secretome, cytoplasm or nuclear extract of HCs and NPCs in response to *in vivo* PB exposure. Proteins found up-regulated in the secretome of HCs are involved in the acute-phase response, inflammation response and the action of drugs. In the cytoplasm of HCs we found 82 proteins up-regulated. These proteins preferentially act in the acute-phase response, cell growth, immune response, inflammatory response as well as in response to cell stress, oxidative stress and wounding. In the nuclear extract of HCs we found 182 proteins up-regulated. These proteins are involved in nucleosome assembly, nucleocytoplasmic transport, mRNA processing, translation, protein localization to the nucleus, RNA processing, removal of superoxide radicals, anti-gen processing and presenting, protein methylation and cell redox homeostasis as well as are major nucleolar proteins of growing eukaryotic cells and proteins of the nuclear matrix. 

In NPCs, 12 proteins were found newly induced, comprising proteins related to inflammation such as PAI-1 and S100-A10, cell migration such as kinesin-1 heavy chain, myosin regulatory light chain RLC-A and dihydropyrimidinase-related protein 1 as well as altered immune cell functional state such as acetyl-CoA carboxylase 1 and ADP-ribosyl cyclase 1. Proteins found up-regulated in the secretome of NPCs are involved in the acute-phase response, inflammation response, and action of drugs and oxidative species. In the cytoplasm of NPCs we found 108 proteins up-regulated. These proteins exert function in tissue, acute phase, cell redox homeostasis, cell cycle, response to drug, cell adhesion, vesicle-mediated protein transport, stress response, positive regulation of MAPKKK cascade, cellular response to cell-matrix adhesion, response to ROS. In the nuclear extract of NPCs we found 78 proteins up-regulated which act on transcription regulation, mRNA processing, mRNA and protein transport, response to DNA damage stimulus and DNA repair.

Table *4* and *5* depict selected proteins found only in untreated rats, which means a down-regulation of these proteins in HCs and NPCs in response to *in vivo* PB exposure. This rather stringent selection criterion for down-regulated proteins improves the reliability of these results. In HCs, 6 proteins were found only in untreated rats, including the E3 ubiquitin-protein ligase UBR4, a protein involved in in membrane morphogenesis and cytoskeletal organization as well as the glutamine synthetase, which catalyses the production of glutamine and 4-aminobutanoate (gamma-aminobutyric acid, GABA). In NPCs, 28 proteins were found only in untreated rats, comprising proteins involved in cell adhesion, cell proliferation, liver development and protein transport.

Table *S1* and *S2* present the summarised proteome profiling results obtained with HCs (S1) and NPCs (S2) derived from PB treated rats (in vivo).

### Pathway analysis of the in vivo data via Reactome

The alterations in the proteome were further analysed by Reactome, a public peer-reviewed pathway database from CSHL, OICR and EBI, which is cross-referenced to bioinformatics databases and allows the assignment of a given protein to one or more molecular pathways. When searching for the positively PB-induced proteome alterations, in both HCs and NPCs, proteins assigned to the categories of “gene expression”, “metabolism of proteins” and “3`UTR-mediated translational regulation” were most prominent among the top listed events (see table *6* & *7*). With respect to molecular pathways, in HCs “Peroxisomal lipid metabolism”, “Class I MHC mediated antigen processing & presentation” and “Asparagine N-linked glycosylation” were listed on top (see table *8*). In contrast, in NPCs “Protein folding”, “Dissolution of Fibrin Clot” and “Platelet Adhesion to exposed collagen” were listed on top (see table *9*).

**Table 6:** Events represented by up-regulated proteins found in HCs upon PB treatment of rats.

**name of the event**	**un-adjusted probability of seeing N or more genes in this event by chance**	**number of genes in the query which map to this event**	**total number of genes involved in this event**
Metabolism	7.40E-17	62	1033
Gene Expression	3.40E-03	25	654
Metabolism of proteins	1.90E-10	26	283
3' -UTR-mediated translational regulation	4.60E-11	19	134
Signal Recognition (Preprolactin)	1.40E-08	15	112
Signal Recognition (Preproinsulin)	1.60E-08	15	113
DNA Replication	2.80E-01	7	241
Cell Cycle	3.20E-01	11	422
Apoptosis	2.00E-02	9	185
Signal Transduction	1.00E+00	14	1710
Cdc20:Phospho-APC/C mediated degradation of Cyclin A	2.50E-03	7	85
Developmental Biology	9.60E-01	5	418
Immune System	9.30E-01	4	319
Membrane Trafficking	1.70E-02	5	69

This indicates effects on molecular events via positively PB-induced proteome alterations.

**Table 7:** Events represented by up-regulated proteins found in NPCs upon PB treatment of rats.

**name of the event**	**un-adjusted probability of seeing N or more genes in this event by chance**	**number of genes in the query which map to this event**	**total number of genes involved in this event**
Metabolism of proteins	4.90E-08	20	283
Gene Expression	7.90E-03	20	654
3' -UTR-mediated translational regulation	2.80E-06	12	134
Metabolism	1.60E-04	33	1033
Signal Recognition (Preprolactin)	1.30E-04	9	112
Signal Recognition (Preproinsulin)	1.40E-04	9	113
Apoptosis	3.30E-04	11	185
DNA Replication	2.50E-01	6	241
Cell Cycle	3.20E-01	9	422
Signal Transduction	1.00E+00	18	1710
Cdc20:Phospho-APC/C mediated degradation of Cyclin A	3.50E-03	6	85
Developmental Biology	1.90E-01	10	418
Muscle contraction	1.10E-01	3	66
Membrane Trafficking	1.20E-01	3	69
Cell-Cell communication	6.80E-01	2	133

**Table 8:** Molecular pathways represented by up-regulated proteins found in HCs upon PB treatment of rats.

**Pathway name**	**Total number of proteins**	**Matching proteins in data**	**% in data**
Peroxisomal lipid metabolism	21	4	19%
Class I MHC mediated antigen processing & presentation	16	3	18%
Asparagine N-linked glycosylation	24	3	12%
Bile acid and bile salt metabolism	27	3	11%
Eukaryotic Translation Elongation	109	12	11%
Eukaryotic Translation Initiation	145	16	11%
Eukaryotic Translation Termination	104	12	11%
RAF/MAP kinase cascade	10	1	10%
SRP-dependent cotranslational protein targeting to membrane	129	14	10%
Translation	178	18	10%
Platelet Adhesion to exposed collagen	11	1	9%
Phase II conjugation	67	6	8%
Biological oxidations	149	11	7%
Lipid digestion, mobilization, and transport	26	2	7%
Membrane Trafficking	69	5	7%
Metabolism of proteins	283	21	7%
Processing of Capped Intronless Pre-mRNA	14	1	7%
Metabolism of amino acids and derivatives	194	12	6%
Fatty acid, triacylglycerol, and ketone body metabolism	90	5	5%
Formation of Fibrin Clot (Clotting Cascade)	36	2	5%
Metabolism of non-coding RNA	19	1	5%
Phase 1 - Functionalization of compounds	84	5	5%
Regulation of Apoptosis	79	4	5%
Signaling by Wnt	69	4	5%

PB treatment of rats may exert a perturbation or up-regulation of the pathways, listed in this table, in HCs. Total number of proteins states the number of different proteins present in the pathway in this database. The matching column gives the proteins number of the number of different proteins also found in our data. The last column shows the calculated percentage value of the previous two columns.

**Table 9:** Molecular pathways represented by up-regulated proteins found in HCs upon PB treatment of rats.

**Pathway name**	**Total number of proteins**	**Matching proteins in data**	**% in data**
Protein folding	35	5	14%
Dissolution of Fibrin Clot	10	1	10%
Platelet Adhesion to exposed collagen	11	1	9%
Signaling by Wnt	69	6	8%
Asparagine N-linked glycosylation	24	2	8%
Lipid digestion, mobilization, and transport	26	2	7%
Regulation of Apoptosis	79	6	7%
Regulation of DNA replication	87	6	6%
Eukaryotic Translation Termination	104	7	6%
Fatty acid, triacylglycerol, and ketone body metabolism	90	6	6%
Eukaryotic Translation Elongation	109	7	6%
Class I MHC mediated antigen processing & presentation	16	1	6%
Rap1 signalling	16	1	6%
Eukaryotic Translation Initiation	145	9	6%
SRP-dependent cotranslational protein targeting to membrane	129	8	6%
APC/C-mediated degradation of cell cycle proteins	100	6	6%
Regulation of mitotic cell cycle	100	6	6%
Metabolism of nucleotides	69	4	5%
Metabolism of amino acids and derivatives	194	11	5%
Metabolism of proteins	283	16	5%
Translation	178	10	5%
Signal amplification	18	1	5%
Semaphorin interactions	79	4	5%
Synthesis of DNA	120	6	5%

PB treatment of rats may exert a perturbation or up-regulation of the pathways, listed in this table, in NPCs. (description see table 6).

### ELISA verification of arginase-1 variations

To verify selected LC-MS/MS data in a quantitative fashion, we conducted an ELISA for rat arginase-1. Arginase-1 was chosen, because of its ability to diminish anti-tumour immunity by interfering with the activation of T-cells [50]. LC-MS/MS results indicated a PB induced decrease in protein secretion of arginase-1 by HCs in case of *in vivo* treated rats. The ELISA results confirmed our LC-MS/MS results as demonstrated in Figure *S1*. In the secretome arginase-1 concentration was found decreased by a factor of 2.3. In the cytoplasmic protein fraction, arginase-1 abundance was found induced by PB more than three-fold, which corresponds very well to the LC-MS/MS results.

## Discussion

The aim of this study was to investigate molecular mechanisms induced by treatment of rats with the non-genotoxic carcinogen phenobarbital (PB) by means of proteome profiling. This approach was used to pinpoint crucial events not previously recognised by other technical approaches. Current mechanistic considerations on non-genotoxic carcinogenesis include altered cell-cell interactions, epigenetic changes endocrine effects, inhibition of gap junctional intercellular communications and immune modulation [6], which may be of crucial importance for initiation as well as promotion and progression [10]. However, the significance of an altered epithelial-mesenchymal dialogue and the role of NPCs for NGC-driven hepatocarcinogenesis have not been investigated so far.

Teufehlofer et al has described that chemical compounds, including genotoxic hepatocarcinogens, may induce the superoxide radical production by Kupffer cells and may thus contribute to DNA damage and an increased occurrence of mutations [51]. Laskin et al and Roberts et al have found that NGCs, such as PB and peroxisome proliferators, may also activate Kupffer cells to release the pro-inflammatory cytokine TNFa [12,25]. Furthermore, stromal liver cells may be induced to secrete survival factors, which may act as tumour promoters [52,53]. Therefore, an improved understanding of non-genotoxic compounds has to consider drug effects on stroma cells, even if these cells are not transformed to cancer cells. In order to assign molecular events caused by NGCs to the different cell types of the liver, we isolated primary cells by liver perfusion and separated them into parenchymal HCs and NPCs as described previously [54,55]. Here, we isolated cells from untreated and PB-treated animals in order to investigate *in vivo* effects.

**Figure 3:**
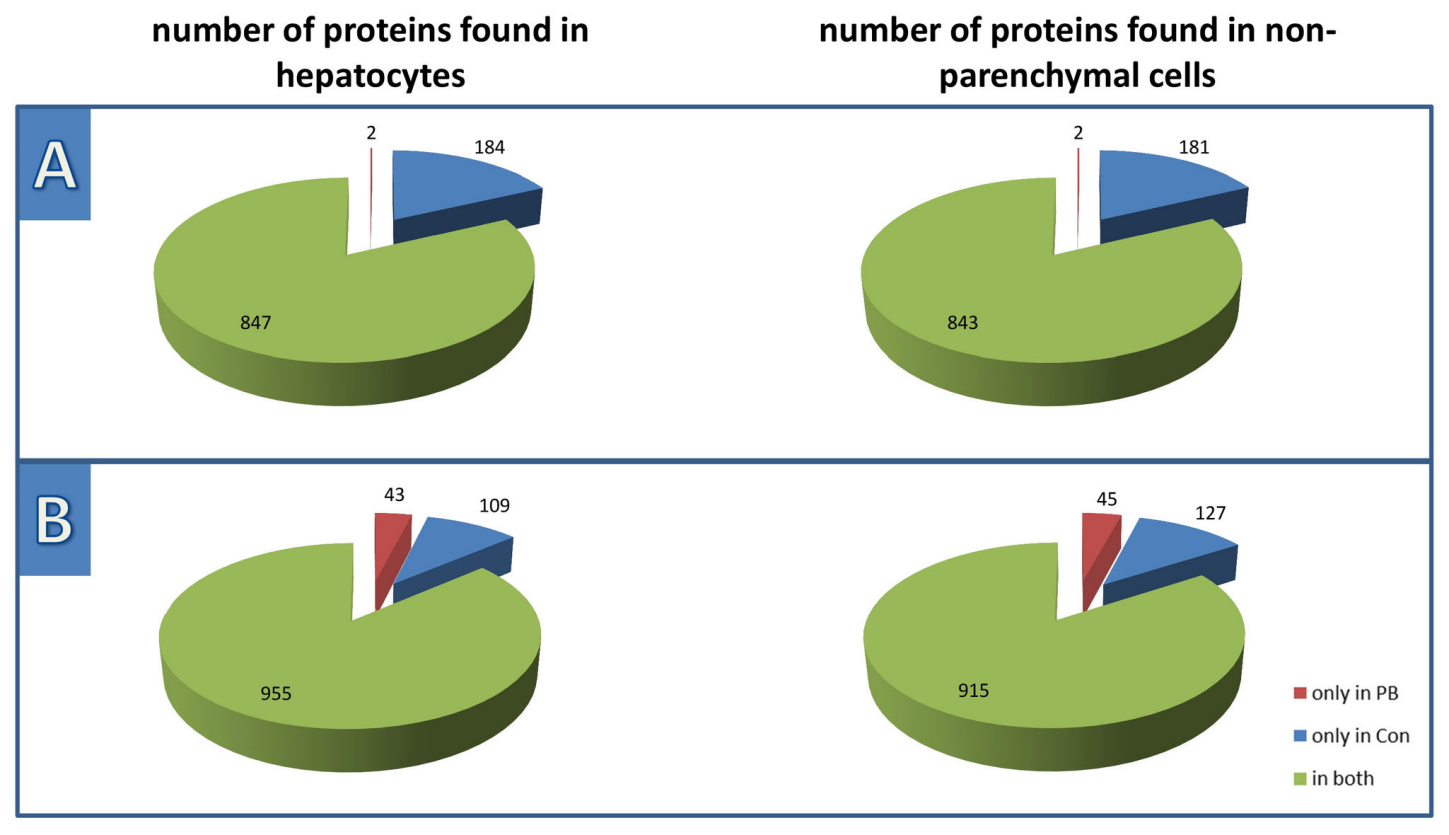
Distribution of distinct proteins, when comparing controls with PB-treatment from the *in* *vitro* and *in* *vivo* sample pools, respectively. This figure demonstrates the distribution of distinct proteins found in HCs and NPCs during the pooled A) *in* *vitro* and B) *in* *vivo* experiments, while including only proteins found with at least 2 peptides. The up- and down-regulation of proteins were neglected in this qualitative comparison.

Cells obtained from untreated livers were also treated with PB *in vitro*. This experimental approach is limited mainly due to the de-differentiation of primary cells during prolonged *in vitro* cultivation. To avoid this issue, we chose a 24 hours treatment period to ensure a meaningful data interpretation. In the *in vitro* part, functional activation by interleukin-6 or LPS resulted in well detectable proteome alterations in HCs or NPCs (data not shown). Any cytotoxic substance will cause a cell stress response, which results in an increased expression of chaperones, especially the heat shock protein family. Under the present experimental conditions PB application *in vitro* hardly induced such a stress response and generally exerted marginal effects. A reason for that may be that the dedifferentiation of HCs in culture during the first 24 hours hampers the reaction of HCs to PB, namely the induction of drug metabolising enzymes. Nevertheless, proteins induced *in vitro* were also found to be induced in the *in vivo* experiments, some of which are presented in Figure *2A*. Figure *3* highlights the diverse response of HCs and NPCs upon *in vitro* (A) and *in vivo* (B) PB treatment. This observation suggests that *in vivo* PB treatment has effects being more profound and largely different from those obtained by *in vitro* treatment. This may be due to the fact that the milieu in the intact organism is required to enable the full response of liver cells to NGCs and that the disrupted cell-cell interactions *in vitro* and the tendency towards de-differentiation under artificial culture conditions compromise such a response.

The presently observed PB-induced proteome alterations in the stromal cells of the liver indeed suggest profound functional alterations. There was strong induction of pleckstrin, which is a positive regulator of platelets causing an aggregation of these sensitive cells, indicating that PB may trigger a wound healing cascade. ADP-ribosyl cyclase 1 (CD38) is an immunity-related protein induced in NPCs upon PB action. This protein usually regulates B cell function by influencing the intra-cellular Ca^2+^ concentration [56]. It was also demonstrated that it affects the migration capabilities of dendritic cells and as a consequence it also affects T cells [57].

The increased secretion of PAI-1, cathepsin L1, MMP-10 and V-CAM 1 indicates some inflammatory activation of these cells. Remarkably, however, a profound inflammatory activation as observed upon LPS-treatment was not evident (Figure *2*). Cox-1 (Q63921), an important mediator of inflammatory signalling, was observed induced by PB in stroma cells in one animal only, which may be due to low protein concentration at the limit of detection. The expression of the inflammation-related protein MX-1 [58] in the control stroma cells, however, indicates that the presently employed cell manipulation steps may also have altered the inflammatory activity state in an artificial way, rendering clear conclusions, with respect to inflammatory pathways, difficult. The present observations still indicate some complex regulatory actions of PB which are related to but still distinct from classical inflammatory activation warranting further investigation.

Many effects of PB on HCs, as observed in our *in vivo* experiments, have already been identified applying techniques other than proteomics, e.g. the induction of phase-I and phase-II drug-metabolising enzymes [59], redox-regulating enzymes as well as the proto-oncogen c-RAF were observed by proteome profiling (table *1*-*3*) [60–62]. As part of the drug metabolism, we found Cytochrome P450 2B1 and other isoforms, sulfotransferase 1A1 and glutathione S-transferase alpha-2 (GST-A2) up-regulated in HCs. Increased glutathione S-transferase levels may indicate increased oxidative stress [63]. It has been proposed that an accumulation of ROS has many effects on cells such as an increased proliferation, DNA mutation rates [64] and genetic instability [65]. Reproducing these and other well-known PB effects by our currently employed proteome profiling analyses supports the notion that this strategy was valid. Furthermore, we were able to observe a few molecular events which have not yet been described. This includes the induction of estradiol 17-beta-dehydrogenase 8 in HCs (table *2*) and the down-regulation of estrogen sulfotransferase 1 in NPCs (table *5*) by PB, which may indicate alterations of the estrogen metabolism. Estrogens are important growth factors and potential tumour promoters [66] and may exert anti-inflammatory activities [67]. Whether PB may act via estrogen activity modulation deserves more detailed investigations, e.g. by including female rats in the PB study. Furthermore, ectonucleotide pyrophosphatase 3 (E-NPP 3) was found to be induced, which has been observed in association with neoplastic bile duct diseases [68].

The nuclear proteins presently observed to be induced by PB actually indicate that cells were finally exposed to DNA stress. This interpretation is supported by the PB-induced expression of DNA topoisomerase I, poly [ADP-ribose] polymerase 1 and sister chromatid cohesion protein PDS5 homolog B. These proteins are known to be involved in DNA damage sensing as well as DNA-repair. These findings were somewhat unexpected, as PB is known to be non-genotoxic. However, it may increase the hepatocellular production of ROS by Cyp450 induction or ROS production by activated NPCs and may thus evoke some marginal DNA repair activity. This demonstrates the potential difficulties to group chemical compounds unequivocally to the categories of genotoxic or non-genotoxic carcinogen. 

Interestingly it was demonstrated that inflammation-related ROS formation and signalling may lead to carcinogenesis of epithelial cells and a phenotypic change. This includes dissolution of cell-cell contacts, cytoplasmic redistribution of E-cadherin and up-regulation of integrins, as evidenced in the present experiments by the up-regulation of integrin-linked protein kinase and matrix metalloproteinases such as MMP-10. Furthermore, under these conditions MMP activity may correlate with invasiveness as determined by Matrigel invasion assays [69]. An PB-induced formation of ROS is evidenced by the observed increase of ROS responding enzymes such as retinal dehydrogenase 1 and mitochondrial superoxide dismutase [Mn] in HCs and plasminogen activator inhibitor 1 (PAI-1) in NPCs. as well as by the induction of DNA repair proteins described above. 

The present investigation of primary cells by proteome profiling may be considered as essential for the achievement of biological relevance of the employed model, but on the other hand, also accounts for the experimental limitation of the present study with respect to reliable quantification of potential marker proteins. Shotgun proteomics as presently employed using nano liquid chromatography and ion trap mass spectrometry may give a quite comprehensive overview to cell activities, but results in rather semi-quantitative data hardly accessible to stringent statistical analysis. However, the present observation of PB-induced proteome alterations especially in NPCs is a first but important step to improve our understanding of the complex mode of action of non-genotoxic carcinogens. 

It is evident from the current results that the analysis of drug effects on isolated cell model systems will hardly represent the mode of action *in vivo*. Furthermore, the contribution of tumour-promoting molecules associated with ROS to the effect of PB is clearly evidenced by the induction of several marker proteins signifying oxidative stress and associated DNA damage. Only the complete comprehension of direct and indirect consequences will enable a strategy to identify a panel of marker molecules with sufficient specificity for the unequivocal indication of non-genotoxic carcinogen activity.

## Supporting Information

Figure S1 **ELISA verification of arginase-1 variations.** This figure depicts the arginase-1 variations in 1) secretome and 2) cytoplasm of HCs using the quantitative data from the ELISA and 3) the semi-quantitative data from the LC-MS/MS analyses (description of how to read this sort of presentation see figure 2), which are altered accordingly. Arginase-1 concentration decreases in the secretome and increases upon PB treatment of rats. Part 4) presents the values used to generate these figure, whereby the emPAI values were used for part 3).
(TIF)

Table S1 **Summary of proteome profiling results obtained with HCs derived from PB treated rats (*in vivo*).** Column names, which are labelled ‘analysis’ and ‘reference’, refer to the experiments of the isolated primary cells, HCs and NPCs, deriving from rats treated *in* *vivo* with PB (analysis) and untreated animals (reference). “found”, specificity of protein identification; accession, Uniprot accession numbers; name, protein names; “analysis_fractions”, subcellular fractions in which the protein was identified in untreated samples; “analysis_peptides”, number of distinct peptides identified per proteins in untreated samples; “analysis_nuclei_expcount”, number of positive identifications in nuclear fractions compared to the total number of experiments; “analysis_nuclei_empai”, calculated emPAI values in the nuclear fraction; “analysis_nuclei_empai_stdev”, standard deviation thereof. These terms are used in the same way referring to the secretome as well as the cytoplasm. After the values obtained for the treated samples (analysis) all values are listed again referring to untreated samples (reference). The last three columns list difference values for the emPAI values obtained for nuclei, secretomes and cytoplasms, respectively.
(XLSX)

Table S2 **Summary of proteome profiling results obtained with NPCs derived from PB treated rats (*in vivo*).** Column names, which are labelled ‘analysis’ and ‘reference’, refer to the experiments of the isolated primary cells, HCs and NPCs, deriving from rats treated *in* *vivo* with PB (analysis) and untreated animals (reference). “found”, specificity of protein identification; accession, Uniprot accession numbers; name, protein names; “analysis_fractions”, subcellular fractions in which the protein was identified in untreated samples; “analysis_peptides”, number of distinct peptides identified per proteins in untreated samples; “analysis_nuclei_expcount”, number of positive identifications in nuclear fractions compared to the total number of experiments; “analysis_nuclei_empai”, calculated emPAI values in the nuclear fraction; “analysis_nuclei_empai_stdev”, standard deviation thereof. These terms are used in the same way referring to the secretome as well as the cytoplasm. After the values obtained for the treated samples (analysis) all values are listed again referring to untreated samples (reference). The last three columns list difference values for the emPAI values obtained for nuclei, secretomes and cytoplasms, respectively.
(XLSX)

## References

[1] Vasseur P, Lasne C (2012) OECD Detailed Review Paper (DRP) number 31 on “Cell Transformation Assays for Detection of Chemical Carcinogens”: main results and conclusions. Mutat Res 744: 8–11. doi:10.1016/j.mrgentox.2011.11.007. PubMed: 22120692. 22120692

[2] Kirkland D, Aardema M, Henderson L, Müller L (2005) Evaluation of the ability of a battery of three in vitro genotoxicity tests to discriminate rodent carcinogens and non-carcinogens I. Sensitivity, specificity and relative predictivity. Mutat Res 584: 1–256. doi:10.1016/j.mrgentox.2005.02.004. PubMed: 15979392. 15979392

[3] Kirkland D, Aardema M, Müller L, Makoto H (2006) Evaluation of the ability of a battery of three in vitro genotoxicity tests to discriminate rodent carcinogens and non-carcinogens II. Further analysis of mammalian cell results, relative predictivity and tumour profiles. Mutat Res 608: 29–42. doi:10.1016/j.mrgentox.2006.04.017. PubMed: 16769241. 16769241

[4] Kirkland D, Kasper P, Müller L, Corvi R, Speit G (2008) Recommended lists of genotoxic and non-genotoxic chemicals for assessment of the performance of new or improved genotoxicity tests: a follow-up to an ECVAM workshop. Mutat Res 653: 99–108. doi:10.1016/j.mrgentox.2008.03.008. PubMed: 18539078. 18539078

[5] Kirkland D, Speit G (2008) Evaluation of the ability of a battery of three in vitro genotoxicity tests to discriminate rodent carcinogens and non-carcinogens III. Appropriate follow-up testing in vivo. Mutat Res 654: 114–132. doi:10.1016/j.mrgentox.2008.05.002. PubMed: 18585956. 18585956

[6] Hernández LG, Van Steeg H, Luijten M, Van Benthem J (2009) Mechanisms of non-genotoxic carcinogens and importance of a weight of evidence approach. Mutat Res 682: 94–109. doi:10.1016/j.mrrev.2009.07.002. PubMed: 19631282. 19631282

[7] Klaunig JE, Babich MA, Baetcke KP, Cook JC, Corton JC et al. (2003) PPARalpha agonist-induced rodent tumors: modes of action and human relevance. Crit Rev Toxicol 33: 655–780. doi:10.1080/713608372. PubMed: 14727734. 14727734

[8] DeLeve LD, Shulman HM, McDonald GB (2002) Toxic injury to hepatic sinusoids: sinusoidal obstruction syndrome (veno-occlusive disease). Semin Liver Dis 22: 27–42. doi:10.1055/s-2002-23204. PubMed: 11928077. 11928077

[9] Phillips JM, Goodman JI (2009) Multiple genes exhibit phenobarbital-induced constitutive active/androstane receptor-mediated DNA methylation changes during liver tumorigenesis and in liver tumors. Toxicol Sci Off J Society Of Toxicology 108: 273–289. doi:10.1093/toxsci/kfp031. PubMed: 19233941. PMC2664694 19233941

[10] Hanahan D, Weinberg RA (2011) Hallmarks of cancer: the next generation. Cell 144: 646–674. doi:10.1016/j.cell.2011.02.013. PubMed: 21376230. 21376230

[11] Bissell MJ, Hines WC (2011) Why don’t we get more cancer? A proposed role of the microenvironment in restraining cancer progression. Nat Med 17: 320–329. doi:10.1038/nm.2328. PubMed: 21383745. 21383745 PMC3569482

[12] Roberts Ra, Ganey PE, Ju C, Kamendulis LM, Rusyn I et al. (2007) Role of the Kupffer cell in mediating hepatic toxicity and carcinogenesis. Toxicol Sci Off J Society Of Toxicology 96: 2–15. doi:10.1093/toxsci/kfl173. PubMed: 17122412. 17122412

[13] Friedman SL (2008). Hepatic Stellate Cells Protean Multifunctional And Enigmatic Cells Liver: 125–172. doi:10.1152/physrev.00013.2007. PMC2888531 18195085

[14] Tanelian DL, Kosek P, Mody I, MacIver MB (1993) The role of the GABAA receptor/chloride channel complex in anesthesia. Anesthesiology 78: 757–776. doi:10.1097/00000542-199304000-00020. PubMed: 8385426. 8385426

[15] Rabow LE, Russek SJ, Farb DH (1995) From ion currents to genomic analysis: recent advances in GABAA receptor research. Synapse (New York, NY) 21: 189–274. doi:10.1002/syn.890210302. PubMed: 8578436. 8578436

[16] Mattson RH, Cramer JA, Collins JF, Smith DB, Delgado-Escueta AV et al. (1985) Comparison of carbamazepine, phenobarbital, phenytoin, and primidone in partial and secondarily generalized tonic-clonic seizures. N Engl J Med 313: 145–151. doi:10.1056/NEJM198507183130303. PubMed: 3925335. 3925335

[17] Perks A, Cheema S, Mohanraj R (2012) Anaesthesia and epilepsy. Br J Anaesth 108: 562–571. doi:10.1093/bja/aes027. PubMed: 22408271. 22408271

[18] Cohen SM, Klaunig J, Meek ME, Hill RN, Pastoor T et al. (2004) Evaluating the human relevance of chemically induced animal tumors. Toxicol Sci Off J Society Of Toxicology 78: 181–186. doi:10.1093/toxsci/kfh073. PubMed: 14737005. 14737005

[19] Kawamoto T, Sueyoshi T, Zelko I, Moore R, Washburn K et al. (1999) Phenobarbital-responsive nuclear translocation of the receptor CAR in induction of the CYP2B gene. Mol Cell Biol 19: 6318–6322. PubMed: 10454578. 10454578 10.1128/mcb.19.9.6318 PMC84602

[20] Yamamoto Y, Moore R, Goldsworthy TL, Negishi M, Maronpot RR (2004) The orphan nuclear receptor constitutive active/androstane receptor is essential for liver tumor promotion by phenobarbital in mice. Cancer Res 64: 7197–7200. doi:10.1158/0008-5472.CAN-04-1459. PubMed: 15492232. 15492232

[21] Phillips JM, Burgoon LD, Goodman JI (2009) The constitutive active/androstane receptor facilitates unique phenobarbital-induced expression changes of genes involved in key pathways in precancerous liver and liver tumors. Toxicol Sci Off J Society Of Toxicology 110: 319–333. doi:10.1093/toxsci/kfp108. PubMed: 19482888. PMC2708600 19482888

[22] Willson TM, Kliewer S a (2002) PXR, CAR and drug metabolism. Nat Rev Drug Discov 1: 259–266. doi:10.1038/nrd753. PubMed: 12120277. 12120277

[23] Handschin C, Ursa Meyer (2003) Induction of Drug Metabolism : The Role of Nuclear. Receptors. 55: 649–673. doi:10.1124/pr.55.4.2.649. 14657421

[24] Elrick MM, Kramer Ja, Alden CL, Blomme E a G, Bunch RT, et al (2005) Differential display in rat livers treated for 13 weeks with phenobarbital implicates a role for metabolic and oxidative stress in nongenotoxic carcinogenicity. Toxicol Pathol 33: 118–126. doi:10.1080/01926230590888298. PubMed: 15805063. 15805063

[25] Laskin DL, Robertson FM, Pilaro AM, Laskin JD (1988) Activation of liver macrophages following phenobarbital treatment of rats. Hepatology (Baltimore, Md) 8: 1051–1055. doi:10.1002/hep.1840080512. PubMed: 2971014. 2971014

[26] Waxman DJ, Azaroff L (1992) Phenobarbital induction of cytochrome P-450. 281: 577–592 10.1042/bj2810577 PMC1130728 1536639

[27] Schwarz M, Peres G, Buchmann A, Friedberg T, Waxman DJ et al. (1987) Phenobarbital induction of cytochrome P-450 in normal and preneoplastic rat liver: comparison of enzyme and mRNA expression as detected by immunohistochemistry and in situ hybridization. Carcinogenesis 8: 1355–1357. doi:10.1093/carcin/8.9.1355. PubMed: 3621474. 3621474

[28] Grivennikov SI, Greten FR, Karin M (2010) Immunity, inflammation, and cancer. Cell 140: 883–899. doi:10.1016/j.cell.2010.01.025. PubMed: 20303878. 20303878 PMC2866629

[29] Holsapple MP, Pitot HC, Cohen SM, Cohen SH, Boobis AR et al. (2006) Mode of action in relevance of rodent liver tumors to human cancer risk. Toxicol Sci Off J Society Of Toxicology 89: 51–56. doi:10.1093/toxsci/kfj001. PubMed: 16221960. 16221960

[30] Teeguarden JG, Dragan Y, Pitot HC (2000) Hazard assessment of chemical carcinogens: the impact of hormesis. J Appl Toxicol JAT 20: 113–120. doi:10.1002/(SICI)1099-1263(200003/04)20:2. PubMed: 10715608. 10715608

[31] Parzefall W, Monschau P, Schulte-Hermann R (1989) Induction by cyproterone acetate of DNA synthesis and mitosis in primary cultures of adult rat hepatocytes in serum free medium. Arch Toxicol 63: 456–461. doi:10.1007/BF00316448. PubMed: 2533487. 2533487

[32] Löw-Baselli a, Hufnagl K, Parzefall W, Schulte-Hermann R, Grasl-Kraupp B (2000) Initiated rat hepatocytes in primary culture: a novel tool to study alterations in growth control during the first stage of carcinogenesis. Carcinogenesis 21: 79–86. doi:10.1093/carcin/21.1.79. PubMed: 10607737. 10607737

[33] Smedsrød B, Pertoft H (1985) Preparation of pure hepatocytes and reticuloendothelial cells in high yield from a single rat liver by means of Percoll centrifugation and selective adherence. J Leukoc Biol 38: 213–230. PubMed: 2993459. 2993459 10.1002/jlb.38.2.213

[34] Hoek JB, Pastorino JG (2002) Ethanol, oxidative stress, and cytokine-induced liver cell injury. Alcohol (Fayetteville, NY) 27: 63–68. doi:10.1016/S0741-8329(02)00215-X. PubMed: 12062639. 12062639

[35] Koj A (1996) Initiation of acute phase response and synthesis of cytokines. Biochim Biophys Acta 1317: 84–94. doi:10.1016/S0925-4439(96)00048-8. PubMed: 8950192. 8950192

[36] Van Snick J (1990) Interleukin-6: an overview. Annu Rev Immunol 8: 253–278. doi:10.1146/annurev.iy.08.040190.001345. PubMed: 2188664. 2188664

[37] Fausto N, Campbell JS, Riehle KJ (2006) Liver regeneration. Hepatology (Baltimore, Md) 43: S45–S53. doi:10.1002/hep.20969. 16447274

[38] Castell JV, Gómez-Lechón MJ, David M, Andus T, Geiger T et al. (1989) Interleukin-6 is the major regulator of acute phase protein synthesis in adult human hepatocytes. FEBS Lett 242: 237–239. doi:10.1016/0014-5793(89)80476-4. PubMed: 2464504. 2464504

[39] Takeda K, Kaisho T, Akira S (2003) Toll-like receptors. Annu Rev Immunol 21: 335–376. doi:10.1146/annurev.immunol.21.120601.141126. PubMed: 12524386. 12524386

[40] Janeway CA, Medzhitov R (2002) Innate immune recognition. Annu Rev Immunol 20: 197–216. doi:10.1146/annurev.immunol.20.083001.084359. PubMed: 11861602. 11861602

[41] Hewett JA, Roth RA (1993) Hepatic and extrahepatic pathobiology of bacterial lipopolysaccharides. Pharmacol Rev 45: 382–411. PubMed: 8127918. 8127918

[42] Kopydlowski KM, Salkowski CA, Cody MJ, Van Rooijen N, Major J et al. (1999) Regulation of macrophage chemokine expression by lipopolysaccharide in vitro and in vivo. J Immunol (Baltimore, Md : 1950) 163: 1537–1544. PubMed: 10415057. 10415057

[43] Paik Y-H, Schwabe RF, Bataller R, Russo MP, Jobin C et al. (2003) Toll-like receptor 4 mediates inflammatory signaling by bacterial lipopolysaccharide in human hepatic stellate cells. Hepatology (Baltimore, Md) 37: 1043–1055. doi:10.1053/jhep.2003.50182. PubMed: 12717385. 12717385

[44] Haudek VJ, Slany A, Gundacker NC, Wimmer H, Drach J et al. (2009) Proteome maps of the main human peripheral blood constituents. J Proteome Res 8: 3834–3843. doi:10.1021/pr801085g. PubMed: 19580323. 19580323

[45] Mortz E, Krogh TN, Vorum H, Görg A (2001) Improved silver staining protocols for high sensitivity protein identification using matrix-assisted laser desorption/ionization-time of flight analysis. Proteomics 1: 1359–1363. doi:10.1002/1615-9861(200111)1:11<1359::AID-PROT1359>3.0.CO;2-Q. PubMed: 11922595. 11922595 10.1002/1615-9861(200111)1:11<1359::AID-PROT1359>3.0.CO;2-Q

[46] Slany A, Haudek VJ, Gundacker NC, Griss J, Mohr T et al. (2009) Introducing a new parameter for quality control of proteome profiles: consideration of commonly expressed proteins. Electrophoresis 30: 1306–1328. doi:10.1002/elps.200800440. PubMed: 19382132. 19382132

[47] Nesvizhskii AI, Keller A, Kolker E, Aebersold R (2003) A statistical model for identifying proteins by tandem mass spectrometry. Anal Chem 75: 4646–4658. doi:10.1021/ac0341261. PubMed: 14632076. 14632076

[48] Kudlicki A (2012) The optimal exponent base for emPAI is. PLOS ONE 65: 7: e32339 doi:10.1371/journal.pone.0032339. PMC3293797 22403647

[49] Griss J, Haudek-Prinz V, Gerner C (2011) GPDE: A biological proteomic database for biomarker discovery and evaluation. Proteomics 11: 1000–1004. doi:10.1002/pmic.201000507. PubMed: 21337704. 21337704

[50] Gabrilovich DI, Nagaraj S (2009) Myeloid-derived suppressor cells as regulators of the immune system. Nat Rev Immunol 9: 162–174. doi:10.1038/nri2506. PubMed: 19197294. 19197294 PMC2828349

[51] Teufelhofer O, Parzefall W, Kainzbauer E, Ferk F, Freiler C et al. (2005) Superoxide generation from Kupffer cells contributes to hepatocarcinogenesis: studies on NADPH oxidase knockout mice. Carcinogenesis 26: 319–329. doi:10.1093/carcin/bgh320. PubMed: 15513930. 15513930

[52] Paulitschke V, Kunstfeld R, Mohr T, Slany A, Micksche M et al. (2009) Entering a new era of rational biomarker discovery for early detection of melanoma metastases: secretome analysis of associated stroma cells. J Proteome Res 8: 2501–2510. doi:10.1021/pr8010827. PubMed: 19222175. 19222175

[53] Sagmeister S, Drucker C, Losert A, Grusch M, Daryabeigi A et al. (2008) HB-EGF is a paracrine growth stimulator for early tumor prestages in inflammation-associated hepatocarcinogenesis. J Hepatol 49: 955–964. doi:10.1016/j.jhep.2008.06.031. PubMed: 18929421. 18929421

[54] Teufelhofer O, Parzefall W, Elbling L, Kainzbauer E, Grasl-Kraupp B et al. (2006) Divide and conquer: rat liver tissue proteomics based on the analysis of purified constituents. Electrophoresis 27: 4112–4120. doi:10.1002/elps.200600017. PubMed: 17054093. 17054093

[55] Lorenz O, Parzefall W, Kainzbauer E, Wimmer H, Grasl-Kraupp B et al. (2009) Proteomics reveals acute pro-inflammatory and protective responses in rat Kupffer cells and hepatocytes after chemical initiation of liver cancer and after LPS and IL-6. Proteomics Clin Appl 3: 947–967. doi:10.1002/prca.200800173. PubMed: 21136998. 21136998

[56] Malavasi F, Deaglio S, Funaro A, Ferrero E, Horenstein AL et al. (2008) Evolution and function of the ADP ribosyl cyclase/CD38 gene family in physiology and pathology. Physiol Rev 88: 841–886. doi:10.1152/physrev.00035.2007. PubMed: 18626062. 18626062

[57] Partida-Sánchez S, Goodrich S, Kusser K, Oppenheimer N, Randall TD et al. (2004) Regulation of dendritic cell trafficking by the ADP-ribosyl cyclase CD38: impact on the development of humoral immunity. Immunity 20: 279–291. doi:10.1016/S1074-7613(04)00048-2. PubMed: 15030772. 15030772

[58] Haudek-Prinz VJ, Klepeisz P, Slany A, Griss J, Meshcheryakova A et al. (2012) Proteome signatures of inflammatory activated primary human peripheral blood mononuclear cells. J of Proteomics 76: 150–62 Spec No: 150–162 doi:10.1016/j.jprot.2012.07.012. PubMed: 22813876. 22813876 PMC3509337

[59] Schaefer O, Ohtsuki S, Kawakami H, Inoue T, Liehner S et al. (2012) Absolute quantification and differential expression of drug transporters, cytochrome P450 enzymes, and UDP-glucuronosyltransferases in cultured primary human hepatocytes. Drug Metab Dispos Biol Fate Chem 40: 93–103. doi:10.1124/dmd.111.042275. PubMed: 21979928. 21979928

[60] Jenke HS, Deml E, Oesterle D (1994) C-raf expression in early rat liver tumorigenesis after promotion with polychlorinated biphenyls or phenobarbital. Xenobiotica; Fate Foreign Compd Biol Syst 24: 569–580. doi:10.3109/00498259409043260. 7526561

[61] Matsuda Y, Fukumoto M (2011) Sorafenib: complexities of Raf-dependent and Raf-independent signaling are now unveiled. Med Mol Morphol 44: 183–189. doi:10.1007/s00795-011-0558-z. PubMed: 22179180. 22179180

[62] Dudgeon C, Peng R, Wang P, Sebastiani A, Yu J et al. (2012) Inhibiting oncogenic signaling by sorafenib activates PUMA via GSK3β and NF-κB to suppress tumor cell growth. Oncogene 31: 4848–4858. doi:10.1038/onc.2011.644. PubMed: 22286758. 22286758 PMC3342476

[63] Dolado I, Swat A, Ajenjo N, De Vita G, Cuadrado A et al. (2007) p38alpha MAP kinase as a sensor of reactive oxygen species in tumorigenesis. Cancer Cell 11: 191–205. doi:10.1016/j.ccr.2006.12.013. PubMed: 17292829. 17292829

[64] Toyokuni S Novel aspects of oxidative stress-associated carcinogenesis. Antioxid Redox Signal Avgust: 1373–1377. doi:10.1089/ars.2006.8.1373. PubMed: 16910784. 16910784

[65] Woo Ra, Poon RYC (2004) Activated oncogenes promote and cooperate with chromosomal instability for neoplastic transformation. Genes Dev 18: 1317–1330. doi:10.1101/gad.1165204. PubMed: 15175263. 15175263 PMC420357

[66] Fox EM, Andrade J, Shupnik MA (2009) Novel actions of estrogen to promote proliferation: integration of cytoplasmic and nuclear pathways. Steroids 74: 622–627. doi:10.1016/j.steroids.2008.10.014. PubMed: 18996136. 18996136 PMC2702758

[67] Vegeto E, Benedusi V, Maggi A (2008) Estrogen anti-inflammatory activity in brain: a therapeutic opportunity for menopause and neurodegenerative diseases. Front Neuroendocrinol 29: 507–519. doi:10.1016/j.yfrne.2008.04.001. PubMed: 18522863. 18522863 PMC2630539

[68] Yano Y, Hayashi Y, Sano K, Nagano H, Nakaji M et al. (2004) Expression and localization of ecto-nucleotide pyrophosphatase/phosphodiesterase I-1 (E-NPP1/PC-1) and -3 (E-NPP3/CD203c/PD-Ibeta/B10/gp130(RB13-6)) in inflammatory and neoplastic bile duct diseases. Cancer Lett 207: 139–147. doi:10.1016/j.canlet.2003.11.002. PubMed: 15072822. 15072822

[69] Mori K, Shibanuma M, Nose K (2004) Invasive potential induced under long-term oxidative stress in mammary epithelial cells. Cancer Res 64: 7464–7472. doi:10.1158/0008-5472.CAN-04-1725. PubMed: 15492271. 15492271

