# Supplementary figures and images for: Phenobarbital Induces Alterations in the Proteome of Hepatocytes and Mesenchymal Cells of Rat Livers
Source: PLoS One. 2013 Oct 24;8(10):e76137. doi: 10.1371/journal.pone.0076137 (PMC3812042; doi:10.1371/journal.pone.0076137)

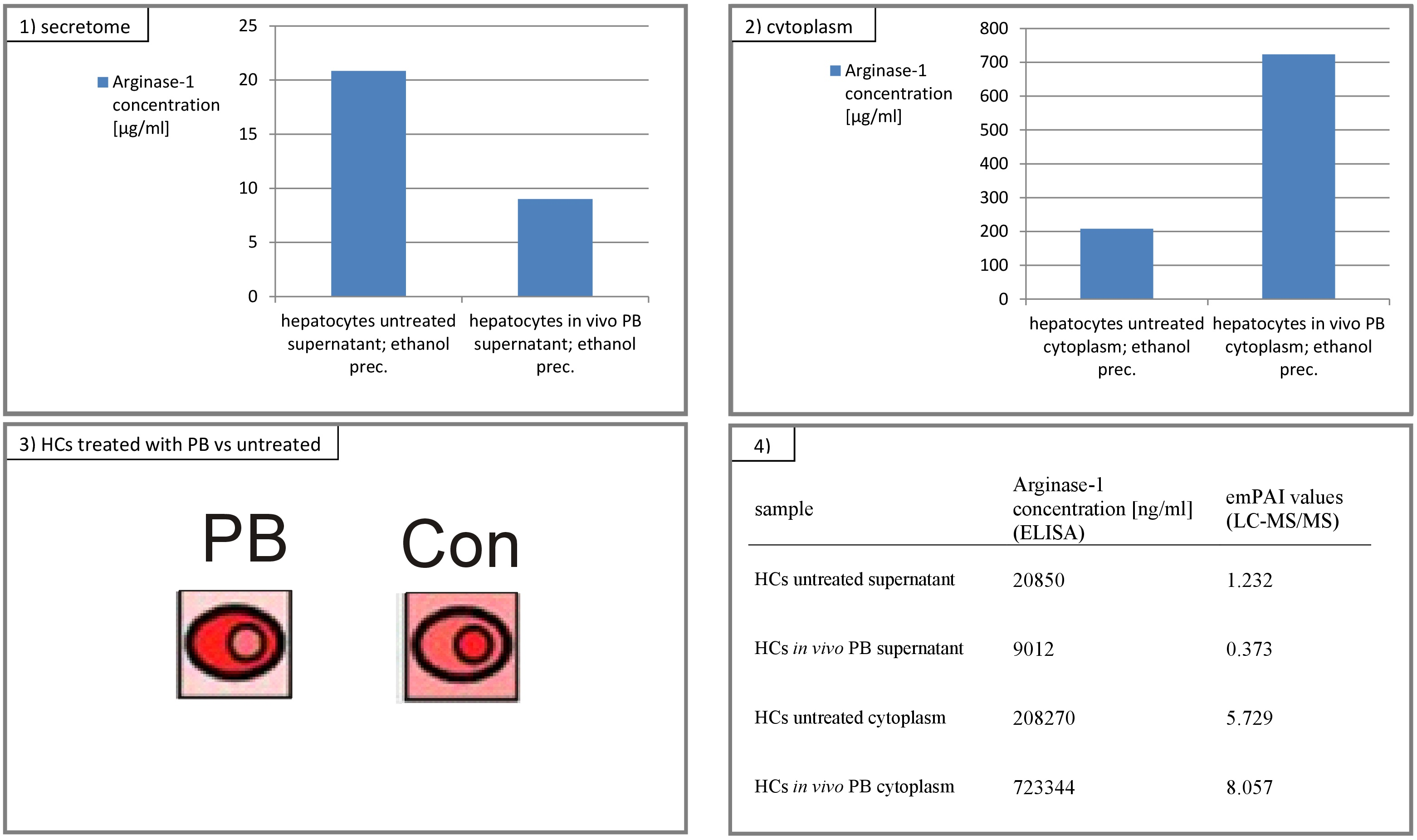

Supplement: Figure S1 — ELISA verification of arginase-1 variations. This figure depicts the arginase-1 variations in 1) secretome and 2) cytoplasm of HCs using the quantitative data from the ELISA and 3) the semi-quantitative data from the LC-MS/MS analyses (description of how to read this sort of presentation see figure 2), which are altered accordingly. Arginase-1 concentration decreases in the secretome and increases upon PB treatment of rats. Part 4) presents the values used to generate these figure, whereby the emPAI values were used for part 3). (TIF) [file pone.0076137.s001.tif]
